# Quantification of experimentally induced nucleotide conversions in high-throughput sequencing datasets

**DOI:** 10.1186/s12859-019-2849-7

**Published:** 2019-05-20

**Authors:** Tobias Neumann, Veronika A. Herzog, Matthias Muhar, Arndt von Haeseler, Johannes Zuber, Stefan L. Ameres, Philipp Rescheneder

**Affiliations:** 1 Research Institute of Molecular Pathology (IMP), Campus-Vienna-Biocenter 1, Vienna BioCenter (VBC), 1030 Vienna, Austria; 2 Institute of Molecular Biotechnology of the Austrian Academy of Sciences (IMBA), Dr. Bohr-Gasse 3, VBC, 1030 Vienna, Austria; 3 Center for Integrative Bioinformatics Vienna, Max F. Perutz Laboratories, University of Vienna, Medical University of Vienna, Dr. Bohrgasse 9, VBC, 1030 Vienna, Austria; 4 Bioinformatics and Computational Biology, Faculty of Computer Science, University of Vienna, Waehringerstrasse 17, A-1090 Vienna, Austria; 5 Medical University of Vienna, VBC, 1030 Vienna, Austria

**Keywords:** Mapping, Epitranscriptomics, Next generation sequencing, High-throughput sequencing

## Abstract

**Background:**

Methods to read out naturally occurring or experimentally introduced nucleic acid modifications are emerging as powerful tools to study dynamic cellular processes. The recovery, quantification and interpretation of such events in high-throughput sequencing datasets demands specialized bioinformatics approaches.

**Results:**

Here, we present Digital Unmasking of Nucleotide conversions in K-mers (DUNK), a data analysis pipeline enabling the quantification of nucleotide conversions in high-throughput sequencing datasets. We demonstrate using experimentally generated and simulated datasets that DUNK allows constant mapping rates irrespective of nucleotide-conversion rates, promotes the recovery of multimapping reads and employs Single Nucleotide Polymorphism (SNP) masking to uncouple true SNPs from nucleotide conversions to facilitate a robust and sensitive quantification of nucleotide-conversions. As a first application, we implement this strategy as SLAM-DUNK for the analysis of SLAMseq profiles, in which 4-thiouridine-labeled transcripts are detected based on T > C conversions. SLAM-DUNK provides both raw counts of nucleotide-conversion containing reads as well as a base-content and read coverage normalized approach for estimating the fractions of labeled transcripts as readout.

**Conclusion:**

Beyond providing a readily accessible tool for analyzing SLAMseq and related time-resolved RNA sequencing methods (TimeLapse-seq, TUC-seq), DUNK establishes a broadly applicable strategy for quantifying nucleotide conversions.

**Electronic supplementary material:**

The online version of this article (10.1186/s12859-019-2849-7) contains supplementary material, which is available to authorized users.

## Background

Mismatches in reads yielded from standard sequencing protocols such as genome sequencing and RNA-Seq originate either from genetic variations or sequencing errors and are typically ignored by standard mapping approaches. Beyond these standard applications, a growing number of profiling techniques harnesses nucleotide conversions to monitor naturally occurring or experimentally introduced DNA or RNA modifications. For example, bisulfite-sequencing (BS-Seq) identifies non-methylated cytosines from cytosine-to-thymine (C > T) conversions [1]. Similarly, photoactivatable ribonucleoside-enhanced crosslinking and immunoprecipitation (PAR-CLIP) enables the identification of protein-RNA-interactions by qualitative assessment of thymine-to-cytosine (T > C) conversions [2]. Most recently, emerging sequencing technologies further expanded the potential readout of nucleotide-conversions in high-throughput sequencing datasets by employing chemoselective modifications to modified nucleotides in RNA species, resulting in specific nucleotide conversions upon reverse transcription and sequencing [3]. Among these, thiol (SH)-linked alkylation for the metabolic sequencing of RNA (SLAMseq) is a novel sequencing protocol enabling quantitative measurements of RNA kinetics within living cells which can be applied to determine RNA stabilities [4] and transcription-factor dependent transcriptional outputs [5] in vitro, or, when combined with the cell-type-specific expression of uracil phosphoribosyltransferase, to assess cell-type-specific transcriptomes in vivo (SLAM-ITseq) [6]. SLAMseq employs metabolic RNA labeling with 4-thiouridine (4SU), which is readily incorporated into newly synthesized transcripts. After RNA isolation, chemical nucleotide-analog derivatization specifically modifies thiol-containing residues, which leads to specific misincorporation of guanine (G) instead of adenine (A) when the reverse transcriptase encounters an alkylated 4SU residue during RNA to cDNA conversion. The resulting T > C conversion can be read out by high-throughput sequencing.

Identifying nucleotide conversions in high-throughput sequencing data comes with two major challenges: First, depending on nucleotide conversion rates, reads will contain a high proportion of mismatches with respect to a reference genome, causing common aligners to misalign them to an incorrect genomic position or to fail aligning them at all [7]. Secondly, Single Nucleotide Polymorphisms (SNPs) in the genome will lead to an overestimation of nucleotide-conversions if not appropriately separated from experimentally introduced genuine nucleotide conversions. Moreover, depending on the nucleotide-conversion efficiency and the number of available conversion-sites, high sequencing depth is required to reliably detect nucleotide-conversions at lower frequencies. Therefore, selective amplification of transcript regions, such as 3′ end mRNA sequencing (QuantSeq [8]) reduces library complexity ensuring high local coverage and allowing increased multiplexing of samples. In addition, QuantSeq specifically recovers only mature (polyadenylated) mRNAs and allows the identification of transcript 3′ ends. However, the 3′ terminal regions of transcripts sequenced by QuantSeq (typically 250 bp; hereafter called 3′ intervals) largely overlap with 3′ untranslated regions (UTRs), which are generally of less sequence complexity than coding sequences [9], resulting in an increased number of multi-mapping reads, i.e. reads mapping equally well to several genomic regions. Finally, besides the exact position of nucleotide conversions in the reads, SLAMseq down-stream analysis requires quantifications of overall conversion-rates robust against variation in coverage and base composition in genomic intervals e.g. 3′ intervals.

Here we introduce Digital Unmasking of Nucleotide-conversions in *k*-mers (DUNK), a data analysis method for the robust and reproducible recovery of nucleotide-conversions in high-throughput sequencing datasets. DUNK solves the main challenges generated by nucleotide-conversions in high-throughput sequencing experiments: It facilitates the accurate alignment of reads with many mismatches and the unbiased estimation of nucleotide-conversion rates taking into account SNPs that may feign nucleotide-conversions. As an application of DUNK, we introduce SLAM-DUNK - a SLAMseq-specific pipeline that takes additional complications of the SLAMseq approach into account. SLAM-DUNK allows to address the increased number of multi-mapping reads in low-complexity regions frequently occurring in 3′ end sequencing data sets and a robust and unbiased quantification of nucleotide-conversions in genomic intervals such as 3′ intervals. SLAM-DUNK enables researchers to analyze SLAMseq data from raw reads to fully normalized nucleotide-conversion quantifications without expert bioinformatics knowledge. Moreover, SLAM-DUNK provides a comprehensive analysis of the input data, including visualization, summary statistics and other relevant information of the data processing. To allow scientists to assess feasibility and accuracy of nucleotide-conversion based measurements for genes and/or organisms of interest in silico, SLAM-DUNK comes with a SLAMseq simulation module enabling optimization of experimental parameters such as sequencing depth and sample numbers. We supply this fully encapsulated and easy to install software package via BioConda, the Python Package Index, Docker hub and Github (see http://t-neumann.github.io/slamdunk) as well as a MultiQC (http://multiqc.info) plugin to make SLAMseq data analysis and integration available to bench-scientists.

## Results

### Digital unmasking of nucleotide-conversions in *k*-mers

**Fig. 1:**
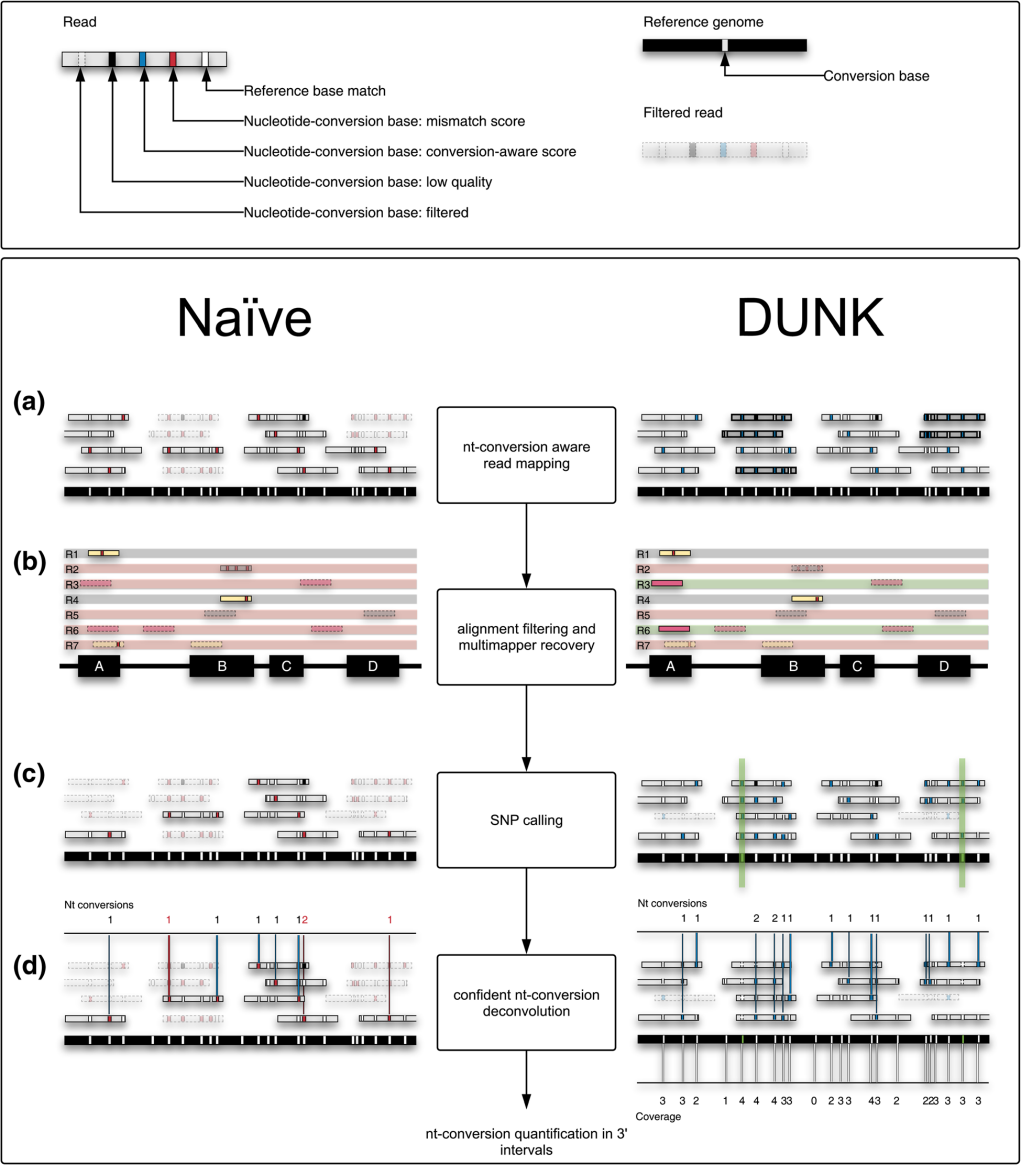
Digital Unmasking of Nucleotide-conversions in *k*-mers: Legend: Possible base outcomes for a given nucleotide-conversion: match with reference (white), nucleotide-conversion scored as mismatch (red), nucleotide-conversion scored with nucleotide-conversion aware scoring (blue), low-quality nucleotide conversion (black) and filtered nucleotide-conversion (opaque) **a** Naïve nucleotide-conversion processing and quantification vs DUNK: The naïve read mapper (left) maps 11 reads (grey) to the reference genome and discards five reads (light grey), that comprise many converted nucleotides (red). The DUNK mapper (right) maps all 16 reads. **b** DUNK processes multi-mapping reads (R5, R6, R7, left) such that the ones (R3, R6) that can be unambiguously assigned to a 3′ interval are identified and assigned to that region, R5 and R7 cannot be assigned to a 3′ interval and will be deleted from downstream analyses. R2 is discarded due to general low alignment quality. **c** False-positive nucleotide conversions originating from Single-Nucleotide Polymorphisms are masked. **d** High-quality nucleotide-conversions are quantified normalizing for coverage and base content

DUNK addresses the challenges of distinguishing nucleotide-conversions from sequencing error and genuine SNPs in high-throughput sequencing datasets by executing four main steps (Fig. 1): First, a nucleotide conversion-aware read mapping algorithm facilitates the alignment of reads (k-mers) with elevated numbers of mismatches (Fig. 1a). Second, to provide robust nucleotide-conversion readouts in repetitive or low-complexity regions such as 3′ UTRs, DUNK optionally employs a recovery strategy for multi-mapping reads. Instead of discarding all multi-mapping reads, DUNK only discards reads that map equally well to two different 3′ intervals. Reads with multiple alignments to the same 3′ interval or to a single 3′ interval and a region of the genome that is not part of a 3′ interval are kept (Fig 1b). Third, DUNK identifies Single-Nucleotide Polymorphisms (SNPs) to mask false-positive nucleotide-conversions at SNP positions (Fig. 1c). Finally, the high-quality nucleotide-conversion signal is deconvoluted from sequencing error and used to compute conversion frequencies for all 3′ intervals taking into account read coverage and base content of the interval (Fig. 1d).

In the following, we demonstrate the performance and validity of each analysis step by applying DUNK to several published and simulated datasets.

### Nucleotide-conversion aware mapping improves nucleotide-conversion quantification

Correct alignment of reads to a reference genome is a central task of most high-throughput sequencing analyses. To identify the optimal alignment between a read and the reference genome, mapping algorithms employ a scoring function that includes penalties for mismatches and gaps. The penalties are aimed to reflect the probability to observe a mismatch or a gap. In standard high throughput sequencing experiments, one assumes one mismatch penalty independent of the type of nucleotide mismatch (standard scoring). In contrast, SLAMseq or similar protocols produce datasets where a specific nucleotide conversion occurs more frequently than all others. To account for this, DUNK uses a conversion-aware scoring scheme (see Table 1). For example, SLAM-DUNK does not penalize a T > C mismatch between reference>read.

**Table 1:** Columns represent reference nucleotide, rows read nucleotide. If a C occurs in the read and a T in the reference, the score is equal to zero. The other possible mismatches receive a score of − 15. A match receives a score of 10

		Reference genome			
		A	T	G	C
Read position	A	10	−15	− 15	− 15
	T	−15	10	−15	− 15
	G	−15	−15	10	−15
	C	−15	0	−15	10

**Fig. 2:**
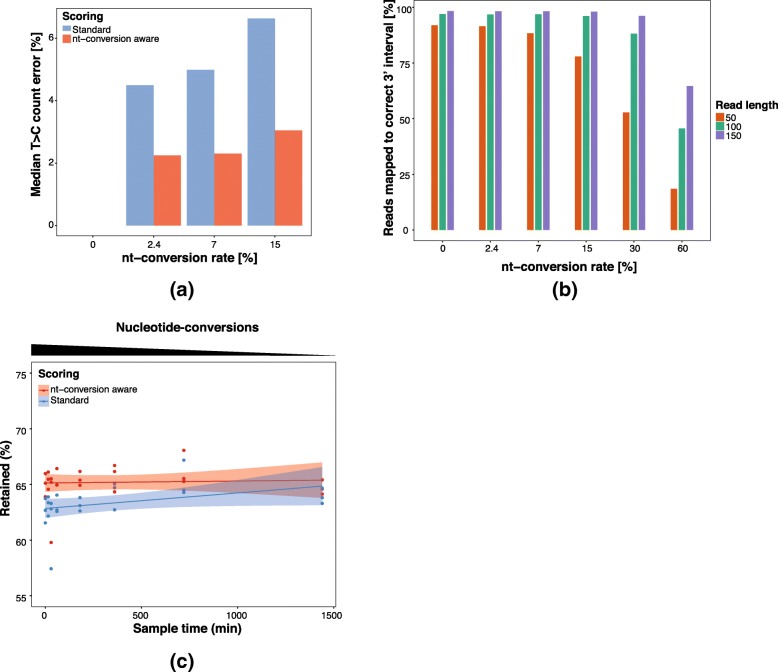
Nucleotide-conversion aware read mapping: **a** Evaluation of nucleotide-conversion aware scoring vs naïve scoring during read mapping: Median error [%] of true vs recovered nucleotide-conversions for simulated data with 100 bp read length and increasing nucleotide-conversion rates at 100x coverage. **b** Number of reads correctly assigned to their 3′ interval of origin for typically encountered nucleotide-conversion rates of 0.0, 2.4 and 7.0% as well as excessive conversion rates of 15, 30 and 60%. **c** Percentages of retained reads and linear regression with 95% CI bands after mapping 21 mouse ES cell pulse-chase time course samples with increasing nucleotide-conversion content for standard mapping and DUNK

We used simulated SLAMseq data with conversion rates of 0% (no conversions), 2.4 and 7% (conversion rates observed in mouse embryonic stem cell (mESC) SLAMseq data [4] and HeLa SLAMseq data (unpublished) upon saturated 4SU-labeling conditions), and an excessive conversion rate of 15% (see Table 2) to evaluate the scoring scheme displayed in Table 1. For each simulated dataset, we compared the inferred nucleotide-conversion sites using either the standard scoring or the conversion-aware scoring scheme to the simulated “true” conversions and calculated the median of the relative errors [%] from the simulated truth (see Methods). For a “conversion rate” of 0% both scoring schemes showed a median error of < 0.1% (Fig. 2a, Additional file 1: Figure S1). Of note, the mean error of the standard scoring scheme is lower than for the conversion-aware scoring scheme (0.288 vs 0.297 nucleotide-conversions) thus favoring standard-scoring for datasets without experimentally introduced nucleotide-conversions. For a conversion rate of 2.4% the standard and the conversion-aware scoring scheme showed an error of 4.5 and 2.3%, respectively. Increasing the conversion rate to 7% further increased the error of the standard scoring to 5%. In contrast, the error of the SLAM-DUNK scoring function stayed at 2.3%. Thus, conversion-aware scoring reduced the median conversion quantification error by 49–54% when compared to standard scoring scheme.

**Table 2:** Simulated datasets and their corresponding analyses in this study

3′ intervals	Nucleotide-conversion rate [%]	Read length [bp]	Coverage	Labeled transcripts	Analysis	Figure
1 k mESC expressed 3′ intervals of 1000 randomly selected transcripts expressed in mESC)	0, 2.4, 7, 15, 30, 60	50, 100, 150	100	100%	Nucleotide-conversion aware read mapping	2a,c, S1
1 k mESC expressed	0, 2.4, 7	50, 100, 150	100	100%	Multimapper recovery strategy evaluation	3b
22,281 (mESC)	0, 2.4, 7	100	60x	100%	SNP masking evaluation	4b
1 k mESC expressed	2.4, 7	50, 100, 150	100x	50%	Evaluation of T > C read sensitivity / specificity	5a, S4
18 example genes (mESC)	2.4, 7	50, 100, 150	200x	50%	Comparison of labeled fraction of transcript estimation methods	5c
1 k mESC expressed	2.4, 7	50, 100, 150	25x- 200x in 25x intervals	0–100%	Evaluation of labeled fraction of transcript estimation	5d, S6

### DUNK correctly maps reads independently of their nucleotide-conversion rate

Mismatches due to SNPs or sequencing errors are one of the central challenges of read mapping tools. Typical RNA-Seq datasets show a SNP rate between 0.1 and 1.0% and a sequencing error of up to 1%. Protocols employing chemically induced nucleotide-conversions produce datasets with a broad range of mismatch frequencies. While nucleotide-conversion free (unlabeled) reads show the same number of mismatches as RNA-Seq reads, nucleotide-conversion containing (labeled) reads contain additional mismatches, depending on the nucleotide-conversion rate of the experiment and the number of nucleotides that can be converted in a read. To assess the effect of nucleotide-conversion rate on read mapping we randomly selected 1000 genomic 3′ intervals of expressed transcripts extracted from a published mESC 3′ end annotation and simulated two datasets of labeled reads with a nucleotide-conversion rate of 2.4 and 7% (see Table 2). Next, SLAM-DUNK mapped the simulated data to the mouse genome and we computed the number of reads mapped to the correct 3′ interval per dataset. Figure 2b shows that for a read length of 50 bp and a nucleotide-conversion rate of 2.4% the mapping rate (91%) is not significantly different when compared to a dataset of unlabeled reads. Increasing the nucleotide-conversion rate to 7% caused a moderate drop of the correctly mapped reads to 88%. This drop can be rectified by increasing the read length to 100 or 150 bp where the mapping rates are at least 96% for nucleotide-conversion rates as large as 15% (Fig. 2b).

While we observe a substantial drop in the percentage of correctly mapped reads for higher conversion rates (> 15%) for shorter reads (50 bp), SLAM-DUNK’s mapping rate for longer reads (100 and 150 bp) remained above 88% for datasets with up to 15 and 30% conversion rates, respectively, demonstrating that SLAM-DUNK maps reads with and without nucleotide-conversion equally well even for high conversion frequencies.

To confirm this finding in real data, we used SLAM-DUNK to map 21 published (7 time points with three replicates each) SLAMseq datasets [4] from a pulse-chase time course in mESCs (see Table 3) with estimated conversion rates of 2.4%. Due to the biological nature of the experiment we expect that the SLAMseq data from the first time point (onset of 4SU- wash-out/chase) contain the highest number of labeled reads while the data from the last time point has virtually no labeled reads.

**Table 3:** Real SLAMseq datasets and their corresponding analyses in this study

Samples	Description	Analysis	Figure
GSM2666819-GSM2666839	Chase-timecourse samples at 0, 0.5,1, 3, 6, 12 and 24 h with 3 replicates at each time point	Percentages of retained reads after mapping with standard and nucleotide-conversion aware scoring with DUNK.	2b
GSM2666816	Single no 4SU 0 h replicate	Multimapper recovery count scatter vs unique mappers	3c, S2b,c, S3
GSM2666816-GSM2666818	3 no 4SU 0 h replicates	Multimapper recovery correlation with RNAseq	3a
GSM2666816-GSM2666821	0 h no 4SU samples and 0 h chase samples with 3 replicates each	Evaluation of SNP calling and masking	4a, c-d
GSM2666816-GSM2666821, GSM2666828-GSM2666837	0 h no 4SU, 0, 3, 6, 12 and 24 h chase samples (3 replicates each)	Evaluation of QC diagnostics	6

Figure 2c shows the expected positive correlation (Spearman’s rho: 0.565, *p*-value: 0.004) between the fraction of mapped reads and the time-points if a conversion unaware mapper is used (NextGenMap with default values). Next, we repeated the analysis using SLAM-DUNK. Despite the varying number of labeled reads in these datasets, we observed a constant fraction of 60–70% mapped reads across all samples (Fig. 2c) and did not observe a significant correlation between the time point and the number of mapped reads (Spearman’s rho: 0.105, *p*-value: 0.625). Thus, DUNK maps reads independent of the nucleotide-conversion rate also in experimentally generated data.

### Multi-mapper recovery increases number of genes accessible for 3’ end sequencing analysis

Genomic low-complexity regions and repeats pose major challenges for read aligners and are one of the main sources of error in sequencing data analysis. Therefore, multi-mapping reads are often discarded to reduce misleading signals originating from mismapped reads: As most transcripts are long enough to span sufficiently long unique regions of the genome, the overall effect of discarding all multi-mapping reads on expression analysis is tolerable (mean mouse (GRCm38) RefSeq transcript length: 4195 bp). By only sequencing the ~ 250 nucleotides at the 3′ end of a transcript, 3′ end sequencing increases throughput and avoids normalizations accounting for varying gene length. As a consequence, 3′ end sequencing typically only covers 3′ UTR regions which are generally of less complexity than the coding sequence of transcripts [9] (Additional file 1: Figure S2a). Therefore, 3′ end sequencing produces a high percentage (up to 25% in 50 bp mESC samples) of multi-mapping reads. Excluding these reads can result in a massive loss of signal. The core pluripotency factor *Oct4* is an example [10]: Although Oct4 is highly expressed in mESCs, it showed almost no mapped reads in the 3′ end sequencing mESC samples when discarding multi-mapping reads (Additional file 1: Figure S3a). The high fraction of multi-mapping reads is due to a sub-sequence of length 340 bp occurring in the *Oct4* 3′ UTR and an intronic region of *Rfwd2.*

**Fig. 3:**
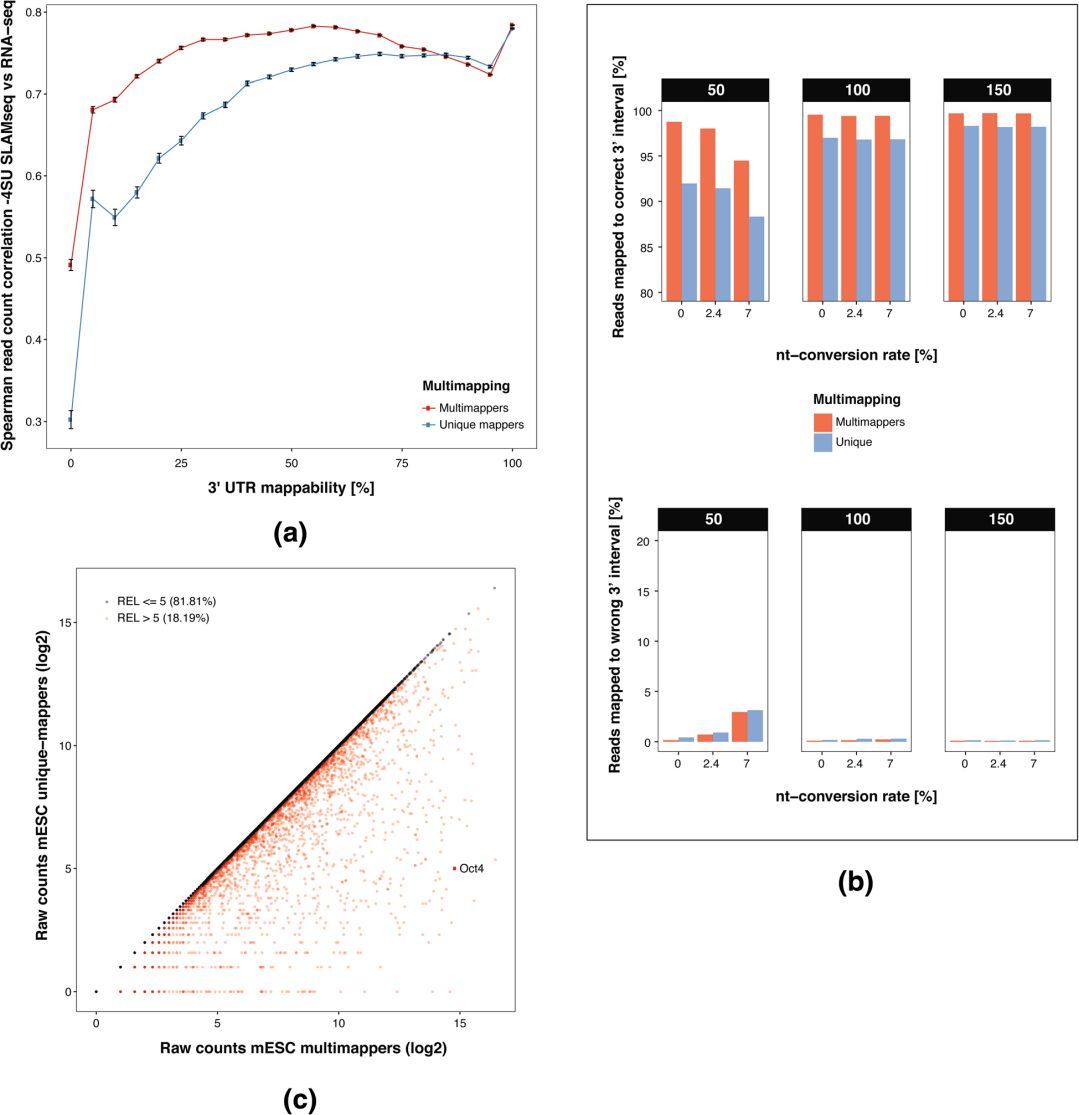
Multimapper recovery strategy in low complexity regions: **a** Correlation of mESC -4SU SLAMseq vs mESC RNA-seq samples (3 replicates each) for unique-mapping reads vs multi-mapping recovery strategy. Spearman mean correlation of all vs all samples is shown for genes with RNAseq tpm > 0 on the y-axis for increasing cutoffs for percentage of unique bp in the corresponding 3′ UTR. Error bars are indicated in black. **b** Percentages of reads mapped to correct (left panel) or wrong (right panel) 3′ interval for nucleotide-conversions rates of 0, 2.4 and 7% and 50, 100 and 150 bp read length respectively, when recovering multimappers or using uniquely mapping reads only **c** Scatterplot of unique vs multi-mapping read counts (log2) of ~ 20,000 3′ intervals colored by relative error cutoff of 5% for genes with > 0 unique and multi-mapping read counts

To assess the influence of low complexity of 3′ UTRs on the read count in 3′ end sequencing, we computed the mappability scores [11] for each 3′ UTR. A high mappability score (ranging from 0.0 to 1.0) of a *k*-mer in a 3′ UTR indicates uniqueness of that k-mer. Next, we computed for each 3′ UTR the %-uniqueness, that is the percentage of its sequence with mappability score of 1. The 3′ UTRs were subsequently categorized in 5% bins according to their %-uniqueness. For each bin we then compared read counts of corresponding 3′ intervals (3 x 4SU 0 h samples, see Table 3) with the read counts of their corresponding gene from a RNA-Seq dataset [4]. Figure 3a shows the increase in correlation as the %-uniqueness increases. If multi-mappers are included the correlation is stronger compared to counting only unique-mappers. Thus, the recovery strategy of multi-mappers as described above efficiently and correctly recovers reads in low complexity regions such as 3′ UTRs. Notably, the overall correlation was consistently above 0.7 for all 3′ intervals with more than 10% of unique sequence.

To further evaluate the performance of the multi-mapper recovery approach, we resorted to simulated SLAMseq datasets: We quantified the percentages of reads mapped to their correct 3′ interval (as known from the simulation) and the number of reads mapped to a wrong 3′ interval, again using nucleotide-conversion rates of 0.0, 2.4 and 7.0% and read lengths of 50, 100 and 150 bp (see Table 2): The multi-mapper recovery approach increases the number of correctly mapped reads between 1 and 7%, with only a minor increase of < 0.03% incorrectly mapped reads (Fig. 3b).

Next, we analysed experimentally generated 3′ end sequencing data (see Table 3) in the nucleotide-conversion free mESC sample. For each 3′ interval, we compared read counts with and without multi-mapper recovery (Fig. 3c). When including multimappers, 82% of the 19,592 3′ intervals changed the number of mapped reads by less than 5%. However, for many of the 18% remaining 3′ intervals the number of mapped reads was highly increased with the multi-mapper-assignment strategy. We found that these intervals show a significantly lower associated 3′ UTR mappability score, confirming that our multi-mapper assignment strategy specifically targets intervals with low mappability (Additional file 1: Figure S2b,c).

Figure 3c also shows the significant increase of the Oct4 read counts when multi-mappers are included (3 x no 4SU samples, mean unique mapper CPM 2.9 vs mean multimapper CPM 1841.1, mean RNA-seq TPM 1673.1, Additional file 1, Figure S3b) and scores in the top 0.2% of the read count distribution. Simulation confirmed that these are indeed reads originating from the Oct4 locus: without multi-mapper assignment only 3% of simulated reads were correctly mapped to *Oct4*, while all reads were correctly mapped when applying multi-mapper recovery.

### Masking single nucleotide polymorphisms improves nucleotide-conversion quantification

Genuine SNPs influence nucleotide-conversion quantification as reads covering a T > C SNP are mis-interpreted as nucleotide-conversion containing reads. Therefore, DUNK performs SNP calling on the mapped reads to identify genuine SNPs and mask their respective positions in the genome. DUNK considers every position in the genome a genuine SNP position if the fraction of reads carrying an alternative base among all reads exceeds a certain threshold (hereafter called variant fraction).

**Fig. 4:**
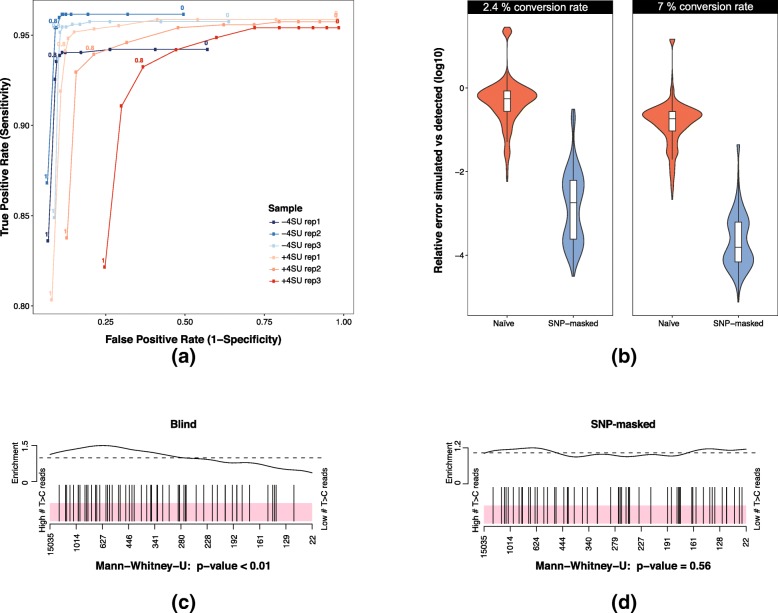
Single-Nucleotide Polymorphism masking: **a** ROC curves for three unlabeled mESC replicates (−4SU) vs three labeled replicates (+4SU) across variant fractions from 0 to 1 in steps of 0.1. **b** Log10 relative errors of simulated T > C vs recovered T > C conversions for naïve (red) and SNP-masked (blue) datasets for nucleotide-conversion rates of 2.4 and 7%. **c** Barcodeplot of 3′ intervals ranked by their T > C read count including SNP induced T > C conversions. Black bars indicate 3′ intervals containing genuine SNPs. **d** Barcodeplot of 3′ intervals ranked by their T > C read count ignoring SNP masked T > C conversions

To identify an optimal threshold, we benchmarked variant fractions ranging from 0 to 1 in increments of 0.1 in three nucleotide-conversion-free mESC QuantSeq datasets (see Table 3). As a ground truth for the benchmark we used a genuine SNP dataset that was generated by genome sequencing of the same cell line. We found that for variant fractions between 0 and 0.8 DUNK’s SNP calling identifies between 93 and 97% of the SNPs that are present in the truth set (sensitivity) (Fig. 4a, −4SU). Note that the mESCs used in this study were derived from haploid mESCs [12]. Therefore, SNPs are expected to be fully penetrant across the reads at the respective genomic position. For variant fractions higher than 0.8, sensitivity quickly drops below 85% consistently for all samples. In contrast, the number of identified SNPs that are not present in the truth set (false positive rate) for all samples rapidly decreases for increasing variant fractions and starts to level out around 0.8 for most samples. To assess the influence of nucleotide-conversion on SNP calling, we repeated the experiment with three mESC samples containing high numbers of nucleotide-conversions (24 h of 4SU treatment). While we did not observe a striking difference in sensitivity between unlabeled and highly labeled replicates, the false-positive rates were larger for low variant fractions suggesting that nucleotide-conversions might be misinterpreted as SNPs when using a low variant fraction threshold. Judging from the ROC curves we found a variant fraction of 0.8 to be a good tradeoff between sensitivity and false positive rate with an average of 94.2% sensitivity and a mean false-positive rate of 16.8%.

To demonstrate the impact of masking SNPs before quantifying nucleotide-conversions, we simulated SLAMseq data (Table 2): For each 3′ interval, we computed the difference between the number of simulated and detected nucleotide-conversions and normalized it by the number of simulated conversion (relative errors) – once with and once without SNP masking (Fig. 4b). The relative error when applying SNP-masking was significantly reduced compared to datasets without SNP masking: With a 2.4% conversion rate, the median relative error dropped from 53 to 0.07% and for a conversion rate of 7% from 17 to 0.002%.

To investigate the effect of SNP masking in real data, we correlated the number of identified nucleotide conversions and the number of genuine T > C SNPs in 3′ intervals. To this end, we ranked all 3′ intervals from the three labeled mESC samples (24 h 4SU labeling) by their number of T > C containing reads and inspected the distribution of 3′ intervals that contain a genuine T > C SNP within that ranking (Fig. 4c and d, one replicate shown). In all three replicates, we observed a strong enrichment (*p*-values < 0.01, 0.02 and 0.06) of SNPs in 3′ intervals with higher numbers of T > C reads (Fig. 4c, one replicate shown). Since T > C SNPs are not assumed to be associated with T > C conversions we expect them to be evenly distributed across all 3′ intervals if properly separated from nucleotide conversions. Indeed, applying SNP-masking rendered enrichment of SNP in 3′ intervals with higher numbers of T > C containing reads not significant (*p*-values 0.56, 0.6 and 0.92) in all replicates (Fig. 4d, one replicate shown).

### SLAM-DUNK: quantifying nucleotide conversions in SLAMseq datasets

The main readout of a SLAMseq experiment is the number of 4SU-labeled transcripts, hereafter called labeled transcripts for a given gene in a given sample. However, labeled transcripts cannot be observed directly, but only by counting the number of reads showing converted nucleotides. To this end, SLAM-DUNK provides exact quantifications of T > C read counts for all 3′ intervals in a sample. To validate SLAM-DUNK’s ability to detect T > C reads, we applied SLAM-DUNK to simulated mESC datasets (for details see Table 2) and quantified the percentage of correctly identified T > C reads i.e. the fraction stemming from a labeled transcript (sensitivity). Moreover, we computed the percentage of reads stemming from unlabeled transcripts (specificity). For a perfect simulation, where all reads that originated from labeled transcripts contained a T > C conversion, SLAM-DUNK showed a sensitivity > 95% and a specificity of > 99% independent of read length and conversion rate (Additional file 1: Figure S4). However, in real datasets not all reads that stem from a labeled transcript contain T > C conversions. To showcase the effect of read length and conversion rate on the ability of SLAMseq to detect the presence of labeled transcripts, we performed a more realistic simulation where the number of T > C conversions per read follows a binomial distribution (allowing for 0 T > C conversions per read).

**Fig. 5:**
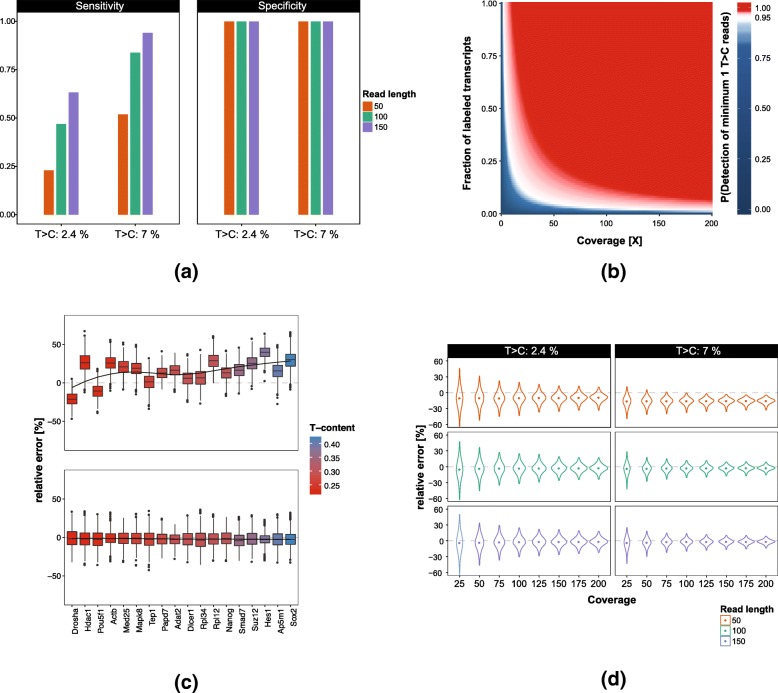
Quantification of nucleotide-conversions: **a** Sensitivity and specificity of SLAM-DUNK on simulated labeled reads vs recovered T > C containing reads for read lengths of 50, 100 and 150 bp and nucleotide-conversion rates of 2.4 and 7%. **b** Heatmap of the probability of detecting at least one read originating from a labeled transcript from a given fraction of labeled transcripts and coverage for a conversion rate of 2.4% and a read length of 50 bp. White color code marks the 0.95 probability boundary. **c** Distribution of relative errors of read-based and SLAM-DUNK’s T-content normalized based *fraction of labeled transcript* estimates for 18 genes with various T-content for 1000 simulated replicates each. **d** Distribution of relative errors of SLAM-DUNK’s T-content normalized *fraction of labeled transcript* estimates for 1000 genes with T > C conversion rates of 2.4 and 7% and sequencing depth from 25 to 200x

As expected, specificity was unaffected by this change (Fig.5a). However, sensitivity changed drastically depending on the read length and T > C conversion rate. While we observed a sensitivity of 94% for 150 bp reads and a conversion rate of 7%, with a read length of 50 bp and 2.4% conversion rate it drops to 23%. Based on these findings we next computed the probability of detecting at least one T > C read for a 3′ interval given the fraction of labeled and unlabeled transcripts for that gene (labeled transcript fraction) for different sequencing depths, read lengths and conversion rates (see Methods) (Fig. 5b, Additional file 1: Figure S5). Counterintuitively, shorter read lengths are superior to longer read lengths for detecting at least one read originating from a labeled transcript, especially for low fractions of labeled transcripts. While 26 X coverage is required for 150 bp reads to detect a read from a labeled transcript present at a fraction of 0.1 and a conversion rate of 2.4%, only 22 X coverage is required for 50 bp reads (Additional file 1: Table S1). This suggests that the higher number of short reads contributes more to the probability of detecting reads from a labeled transcript than the higher probability for observing a T > C conversion of longer reads. Increasing the conversion-rate to 7% reduces the required coverage by ~ 50% across fractions of labeled transcripts, again with 50 bp read lengths profiting most from the increase. In general, for higher labeled transcript fractions such as 1.0 the detection probability converges for all read lengths to a coverage of 2–3 X and 1 X for conversion rates of 2.4 and 7%, respectively (Additional file 1: Figure S5). Although, these results are a best-case approximation they can serve as guideline on how much coverage is required when designing a SLAMseq experiment that relies on T > C read counts to detect labeled transcripts.

While estimating the number of labeled transcripts from T > C read counts is sufficient for experiments comparing the same genes in different conditions and performing differential gene expression-like analyses, it does not account for different abundancies of total transcripts when comparing different genes. To address this problem, the number of labeled transcripts for a specific gene must be normalized by the total number of transcripts present for that gene. We will call this the *fraction of labeled transcripts*. A straight forward approach to estimate the *fraction of labeled transcripts* is to compare the number of labeled reads to the total number of sequenced reads for a given gene (see Methods). However, this approach does not account for the number of Uridines in the 3′ interval. Reads originating from U-rich transcript or a T-rich part of the corresponding genomic 3′ interval have a higher probability of showing a T > C conversion. Therefore, T > C read counts are influenced by the base composition of the transcript and the coverage pattern. Thus, the *fraction of labeled transcripts* will be overestimated for T-rich and underestimated for T-poor 3′ intervals. To normalize for the base composition, SLAM-DUNK implements a T-content and read coverage normalized approach for estimating the *fractions of labeled transcripts* (see Methods). To evaluate both approaches we picked 18 example genes with varying T content in their 3′ intervals, 3′ interval length and mappability (see Additional file 1: Table S2 for full list), simulated 1000 SLAMseq datasets (see Table 2) for each gene and compared the recovered *fraction of labeled transcripts* with the simulated truth (Fig. 5c). On average the read-count based method showed a mean relative error of 15%. In contrast, SLAM-DUNK’s T-content normalized approach showed a mean relative error of only ~ 2%. Inspection of the 18 genes revealed high variability in the estimates of the read-count based method. While both methods perform equally well for *Tep1*, the median error of the other 17 genes varies between 6 and 39% for the read-based method and only between 1 and 4% for SLAM-DUNK. We observed a strong correlation of relative error and T-content using the read-count based method (Pearson’s r: 0.41) and only a very weak association when using SLAM-DUNK’s T-content normalized approach (Pearson’s r: − 0.04). Expanding the analysis from 18 to 1000 genes confirmed the result. For the T > C read-based approach, 23% of the 3′ intervals showed a relative error larger 20%. For SLAM-DUNK’s T-content normalized approach it was only 8%.

Important factors for how confidently we can assess the *fraction of labeled transcripts* of a given gene are the T > C conversion rate, read length and sequencing depth. To assess how much SLAMseq read coverage is required for a given read length, we computed the relative error in *fraction of labeled transcripts* using SLAM-DUNK’s T content normalized approach estimation for datasets with a conversion rate of 2.4 and 7%, read lengths of 50, 100 and 150 bp and sequencing depth of 25 to 200 (Fig. 5d). First, we looked at datasets with a T > C conversion rate of 2.4%. With a read length of 50 bp, SLAM-DUNK underestimated *the fractions of labeled transcripts* by about 10%. This is caused by multi-mapping reads that cannot be assigned to a single 3′ interval. Increasing the read length to 100 or 150 bp allows SLAM-DUNK to assign more reads uniquely to the genome. Therefore, the median relative error is reduced to 3% for these datasets. Sequencing depth showed no influence on the median relative error. However, it influences the variance of the estimates. With a read length of 100 bp and a coverage of 50X, 18% of the 3′ intervals show a relative error of > 20%. Increasing the coverage to 100X or 150X, reduces this number to 6 and 0.8%, respectively.

Increasing the T > C conversion rate to 7% improved overall *fraction of labeled transcripts* estimations noticeably. For 100 bp reads and a coverage of 50X, 100X and 200X the percentage of 3′ intervals with relative error > 20% is reduced to 3, 0.2 and 0%, respectively. Independent of read length, coverage and T > C conversion rate, the T > C read based *fraction of labeled transcripts* estimates performed worse than the SLAM-DUNK estimates (see Additional file 1: Figure S6).

**Fig. 6:**
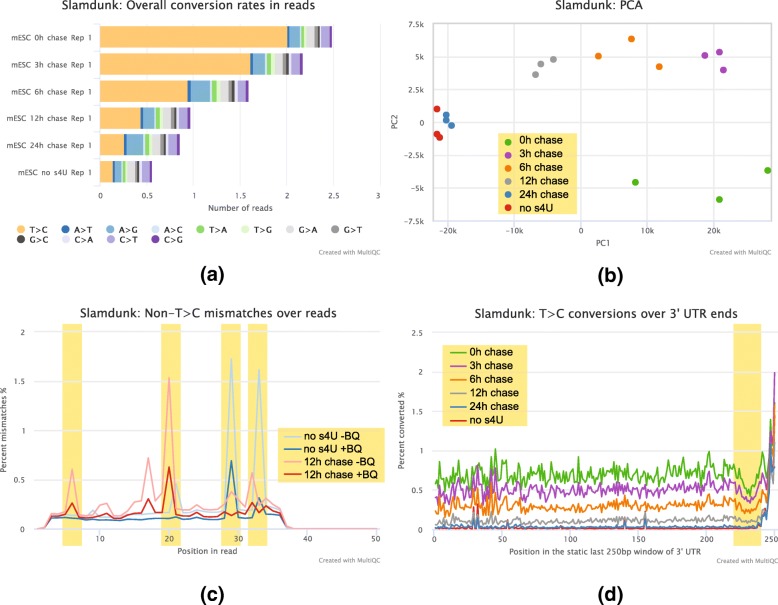
Integrated quality controls: **a** Nucleotide-conversion rates of read sets from 6 representative mESC time courses showing a decrease of T > C conversions proportional to their respective chase time. **b** T > C conversion containing read based PCA of 6 mESC timepoints (3 replicates each). **c** Distribution of non-T > C mismatches across read positions display spikes in error rates (highlighted in yellow) for a low T > C conversion content (no 4SU) and a high T > C conversion (12 h chase) sample which are dampened or eradicated when applying base-quality filtering. **d** Nucleotide-conversion distribution along 3′ end positions in a static 250 bp at 3′ UTR ends for mESC time-course showing characteristic curve shifts according to their presumed T > C conversion content (timepoint) and a strong base conversion bias towards the 3′ end (highlighted in yellow) induced by generally reduced T-content in the last bases of 3′ UTRs

Both *fraction of labeled transcripts* estimates as well as raw T > C read counts are affected by sequencing error, especially when the T > C conversion rate is low. To mitigate the impact of sequencing error on the respective quantification measures, SLAM-DUNK optionally applies a base-quality filter on conversion calls. As shown in Fig. 6c, this strategy substantially reduces the signal from erroneous sequencing cycles. In addition, SLAM-DUNK allows the quantifications of *fraction of labeled transcripts* estimates as well as raw T > C read counts to be restricted to reads that carry > 1 nucleotide-conversions. Muhar et al. [5] showed that using this strategy, the contribution of background signal from reads with 1 T > C conversion was almost completely eradicated when using reads with 2 T > C conversions. Alternatively, the background signal of no 4SU could be subtracted to address sequencing error as performed by Herzog et al. [4].

### Quality control and interpretation of SLAMseq datasets

To facilitate SLAMseq sample interpretation, we implemented several QC modules into SLAM-DUNK on a per-sample basis. To address the need for interpretation of samples in an experimental context, we provide MultiQC support [13] for SLAM-DUNK. SLAM-DUNK’s MultiQC module allows inspection of conversion rates, identification of systematic biases and summary statistics across samples.

To demonstrate SLAM-DUNK’s QA capabilities, we applied it to 6 representative mESC timecourse datasets with expected increasing nucleotide-conversion content (see Table 3). First, we compared the overall nucleotide conversions rates of all timepoints and observed the expected decrease of T > C nucleotide-conversions in later time-points (Fig. 6a, one replicate shown). Next, we performed a PCA based on T > C conversion containing reads using all three replicates. We found that replicates cluster together as expected. Furthermore, 24 h chase and no 4SU samples formed one larger cluster. This can be explained since at 24 h of chase, samples are expected to be T > C conversion free (Fig. 6b).

By inspecting mismatch rates along read positions for two representative samples, we could identify read cycles with increased error rates (Fig. 6c). To reduce read cycle dependent nucleotide mismatch noise, we implemented a base-quality cutoff for T > C conversion calling in SLAM-DUNK. Applying the base-quality cutoffs significantly increased overall data quality, mitigating or even eradicating error-prone read positions. Finally, we visualized average T > C conversion rates across the last 250 nucleotides of each transcript to inspect positional T > C conversion biases across the 3′ intervals. We found no conversion bias across the static 250 bp windows except for a dip in T > C conversions ~ 20 nucleotides upstream of the 3′ end, which is most likely caused by lower genomic T-content, a characteristic feature of mRNA 3′ end sequences (see Additional file 1: Figure S7).

## Discussion and conclusions

We present Digital Unmasking of Nucleotide-conversions in *k*-mers (DUNK) for mapping and quantifying nucleotide conversions in reads stemming from nucleotide-conversion based sequencing protocols. As a showcase application of DUNK, we applied it to T > C nucleotide-conversion containing datasets as produced by the novel SLAMseq protocol. Using real and simulated datasets, we establish DUNK as method that allows nucleotide-conversion rate independent read mapping for nucleotide-conversion rates of up to 15% when analyzing 100 bp reads. Since the most informative proportion of the overall signal stems from nucleotide-conversion containing reads, the correct mapping of such reads is crucial and resulted in a reduction of the nucleotide-conversion quantification error by ~ 50%. DUNK tackles the problem of low-complexity and repetitive sequence content which is severely aggravated in 3′ end sequencing by employing a multi-mapper recovery strategy: We demonstrate that DUNK specifically recovers read mapping signal in 3′ intervals of low mappability that would otherwise be inaccessible to 3′ end sequencing approaches, enhancing the correlation with complementary RNAseq data. Globally, we recover an additional 1–7% of correctly mapped reads at a negligible cost of wrongly mapped reads. We used genome-sequencing datasets to establish optimized variant calling settings, a crucial step of DUNK to separate true- from false-positive nucleotide-conversion stemming from SNPs. Applying these established settings, we demonstrated the advantage of SNP masking over naïve nucleotide-conversion quantification which uncouples SNPs from nucleotide-conversion content and results in a more accurate nucleotide-conversion quantification, reducing the median quantification error for SNP harboring 3′ intervals from 53 to 0.07% and 17 to 0.002% for conversion rates of 2.4 and 7%, respectively.

We provide the SLAM-DUNK package, an application of DUNK to SLAMseq datasets: SLAM-DUNK provides absolute read-counts of T > C conversion containing reads which can directly be used for comparing the same transcripts in different conditions and to perform differential gene expression-like analyses with a sensitivity of 95%. Since absolute-read counts between genes are dependent on T-content and sequencing depth, SLAM-DUNK implements a T-content and read coverage normalized approach for estimating the *fractions of labeled transcripts*. This quantification routine has clear advantages over read-count based *fraction of labeled transcripts* estimates, reducing the proportion of genes with relative errors > 20% from 23% with the read-based approach to only 8% with SLAM-DUNK’s T-content normalized approach. In addition to absolute T > C read counts and *fractions of labeled transcript* estimates, SLAM-DUNKs modular design also allows to plug-in statistical frameworks such as GRAND-SLAM [14] which utilizes a binomial mixture-model for estimating proportions of new and old RNA to directly estimate RNA half-lives.

While SLAM-DUNK is a showcase application of DUNK to unpaired, stranded and unspliced (QuantSeq) data, NextGenMap – our mapper of choice – is a general-purpose alignment tool that also facilitates paired-end, unstranded datasets. Therefore, NextGenMap can be readily parametrized to process datasets produced by novel applications such as NASC-seq (https://www.biorxiv.org/content/early/2018/12/17/498667) and scSLAM-seq (https://www.biorxiv.org/content/10.1101/486852v1) with the downstream SLAM-DUNK pipeline. Due to SLAM-DUNK’s modular design, one can also entirely swap the alignment tool to other standard RNA-seq aligners as long as they output the BAM-tags we introduced for speedy conversion detection (see Additional file 1: Supplementary information).

SLAM-DUNK’s simulation framework allows assessing error rates for given parameters such as read length, conversion rate and coverage in silico prior to setting up experiments in vitro. This allows bench scientists to inspect simulation results to check whether they are able to reliably interpret nucleotide-conversion readouts for given genes using a certain experimental setup and annotation.

Ensuring scalability as well as feasible resource consumptions is vital for processing large multisample experiments such as multi-replicate time courses. SLAM-DUNK achieves this with its modular design and efficient implementation enabling a 21-sample time course experiment to run in under 8 h hours with 10 CPU threads on a desktop machine with a peak memory consumption of 10 GB main memory (see Additional file 1: Supplementary information).

We demonstrated that SLAM-DUNK visualizations and sample-aggregation via MultiQC are valuable tools to unravel biases in and characteristics of SLAMseq datasets thus facilitating rapid and easy quality checks of samples and providing measures to correct for systemic biases in the data.

Deployment of SLAM-DUNK on multiple software platforms and through Docker images ensures low effort installation on heterogeneous computing environments. Verbose and comprehensive output of SLAM-DUNK makes results reproducible, transparent and immediately available to bench-scientists and downstream analysis tools.

## Methods

### Mapping reads with T > C conversions

NextGenMap [7] maps adapter- and poly(A)-trimmed SLAMseq reads to the user specified reference genome. Briefly, NextGenMap searches for seed words - 13-mers that match between a given read and the reference sequence - using an index data structure. All regions of the reference sequence that exceed a certain seed-word count threshold are candidate mapping regions (CMR) for the read. Subsequently, NextGenMap identifies the CMR with the highest pairwise Smith-Waterman alignment score as the best mapping position of the read in the genome. If a read has more than one CMR NextGenMap reports up to 100 locations in the genome. Reads with more than 100 mapping locations are discarded.

We extended NextGenMap’s seed-word identification step to allow for a single T > C mismatch in a seed word. Finally, we changed the scoring function of the pairwise sequence alignment algorithms to assign neither a mismatch penalty nor a match score to T > C mismatches:

Furthermore, we extended NextGenMap to output additional SAM tags containing all necessary information (e.g. list of all mismatches in a read) required by subsequent steps of SLAM-DUNK (see Additional file 1: Supplementary information for details).

### Filtering reads and multi-mapper assignment

Only read alignments with a sequence identity of at least 95% and a minimum of 50% of the read bases aligned were kept for the subsequent analysis. Since 3′ end sequencing should generate fragments at 3′ end mRNAs, we discard all read mappings located outside of a user-defined set of 3′ UTR intervals. Still, remaining multi-mapper reads are processed as follows: For a read that maps to two or more locations of the same 3′ UTR, one location is randomly picked, the others are removed. All reads that map to two or more distinct 3′ UTRs are entirely discarded (see Additional file 1: Figure S8 for details).

### SNP masking

SLAM-DUNK uses VarScan 2.4.1 [15] to call SNPs in the set of filtered reads requiring a minimum coverage of 10x and a minimum alternative allele frequency of 0.8 for all published and sequenced haploid samples. Thus, if VarScan identified a C as a new SNP at a T nucleotide in the genome, this will not be counted as T > C conversion in downstream analysis.

For genome-sequencing data, SNPs were called using VarScan 2.4.1 default parameters only outputting homozygous variant positions. Only SNPs that exceed the minimum coverage of the respective Varscan 2.4.1 runs in the benchmarked 3′ intervals are considered for sensitivity and false-positive rate calculations.

We used an adaption of the *barcodeplot* function of the limma package to visualize the distribution of SNPs along 3′ intervals ordered by their number of T > C reads: To make sure the SNP calls are not coverage biased, we only use the upper quartile of 3′ intervals in terms of read coverage, excluding 3′ intervals not meeting the coverage cutoffs of the variant calling process for this analysis. We produce one plot using unmasked T > C containing reads and a separate plot using SNP-masked T > C containing reads. In addition, we apply the Mann-Whitney-U test on both sets to give a measure of how biased the SNPs are distributed in unmasked vs SNP-masked 3′ intervals. Ideally, a strong association of T > C containing reads with SNPs in the unmasked data and no association of T > C containing reads with SNPs in the masked data is expected, showing the SNP calling actually uncoupled T > C conversions and SNPs. These plots allow to visually assess the quality/performance of SNP calling on the data without presence of actual controls.

### Estimating the fraction of labeled transcripts

Let *p*_*SU*_ be the unknown fraction of labeled transcripts for a 3 ′ interval. With 0 ≤ *p*_*e*_ ≤ 1 we denote the efficiency that a transcript contains a 4SU, the 4SU residue is alkylated and that alkylated 4SU base-pairs with G instead of U during reverse transcription, that can be identified as a T > C conversion in high-throughput sequencing. Note, we assume *p*_*e*_ is constant for a given experiment.

### T > C read counts based estimator

The probability that a read from a labeled transcript does not show a T > C conversion equals

$$ {p}_0={\left(1-{p}_e\right)}^t $$

where *t* is the number of thymidines in the read matching genomic sequence. Accordingly, the probability to find a read with at least one T > C conversion from a labeled transcript equals

$$ {p}_{T\to C}=1-{p}_0 $$

In a sample of *n* reads given an unknown frequency *p*_*SU*_ of labeled transcripts we expect to find

$$ E(SU)={p}_{SU}\ast {p}_{T\to C}\ast n $$

reads with at least one conversion site. Note, we assume that *t* is the same for all reads. Based on the observed number of converted reads *R*_*T→C*_ and the number of reads *n*, we get

$$ \widehat{p_{SU}\ast {P}_{T\to C}}=\frac{R_{T\to C}}{n} $$

In a time course experiment, we can use a time-point with a labeling time long enough that all transcripts are labeled (*p*_*SU*_=1) for all 3' intervals to retrieve *P*_*T→C*_. Since we assume that *P*_*T→C*_ is constant for a given experiment we obtain

$$ \widehat{p_{SU}}=\frac{1}{P_{T\to C}}\ast \frac{R_{T\to C}}{n} $$

for all other time points.

### T-content and coverage normalized estimator

Since assuming *t* to be the same for all reads is an oversimplification we want to estimate *p*_*SU*_ without using T > C read counts by looking at T-positions individually. For a specific T-position *i* in the 3' interval, let *X*_*i*_ denote the number of reads that show a conversion and let *c*_*i*_ define the number of reads that cover position *i*. Then

$$ \Pr \left({X}_i={k}_i\ \right|\ {p}_{SU},{p}_e\Big)=\left({c}_i,{k}_i\right){\left({p}_{SU}\ast {p}_e\right)}^k{\left(1-{p}_{SU}\ast {p}_e\right)}^{\left({c}_i-{k}_i\right)}, $$

If *p*_*e*_ is unknown, we can compute the maximum likelihood estimate of the confounded probability *p*_*SU*_ * *p*_*e*_ as

$$ \widehat{p_{SU}\ast {p}_e}=\frac{k_i}{c_i} $$

If the interval contains *n T*s then the maximum likelihood estimate of *p*_*SU*_ * *p*_*e*_ equals

$$ \widehat{p_{SU}\ast {p}_e}=\frac{\sum_{i=1}^n{k}_i}{\sum_{i=1}^n{c}_i} $$

By retrieving *p*_*e*_ from an experiment with *p*_*SU*_=1 we obtain

$$ \widehat{p_{SU}}=\frac{1}{p_e}\ast \frac{\sum_{i=1}^n{k}_i}{\sum_{i=1}^n{c}_i} $$

### Computing the probability of detecting at least one T > C read for a 3’ interval

Based on the notation developed above, we can compute the expected number of reads showing a T > C conversion given *p*_*SU*_ and *p*_*T→C*_ for a 3' interval with *n* sequenced reads as

$$ E(SU)={p}_{SU}\ast {p}_{T\to C}\ast n $$

When taking into account empirically determined sensitivity of SLAMDUNK *S*, i.e. the probability of detecting a read with T > C conversion as a labeled read, we compute the probability of detecting at least one labeled read for a 3′ interval given *p*_*SU*_ and *p*_*T* → *C*_ as:

$$ {p}_{DETECT}=1-B\left(0;E(SU),S\right) $$

### Simulating SLAMseq datasets

All datasets were simulated using SLAM-DUNK’s simulation module. The input parameters are a reference genome sequence and a BED file containing 3′ interval annotations. First, SLAM-DUNK removes overlapping 3′ intervals in the BED file and assigns a labeled transcript fraction between 0 and 1 (uniformly distributed) to each 3′ interval. Alternatively, the user can either supply a fixed fraction of labeled transcripts that is the same for all genes or add gene specific fraction to the initial BED file. Next, SLAM-DUNK extracts the 3′ interval sequences from the supplied FASTA file and randomly adds homozygous SNPs, including but not limited to T > C SNPs, based on a user specified probability (default: 0.1%). For each of the modified 3′ interval SLAM-DUNK simulates RNA reads using the RNASeqReadSimulator (https://github.com/davidliwei/RNASeqReadSimulator) package. To mimic Quant-Seq datasets, only the last 250 bp of the 3′ interval are used for the simulation. Finally, SLAM-DUNK adds T > C conversions to the reads to simulate transcripts labeling. The number of T > C conversions for each labeled read is computed using a binomial distribution *B(t, p*_*e*_*)* with *t* the number of Ts in the read and *p*_*e*_ the conversion probability. All simulated reads are stored in a BAM file. The name of each read contains the name of the 3′ interval the read was simulated from and the number of T > C conversions added. Furthermore, SLAM-DUNK provides a T > C count file containing the number of simulated reads and simulated fraction of labeled transcripts for all 3′ interval.

### Relative error

Let *N*_*TRUE*_ be the number of true events and *N*_*DETECT*_ the number of detected events. Then the relative error *E*_*rel*_ of a detected quantity compared to the known truth calculates as follows:

$$ {E}_{rel}=\frac{N_{TRUE}-{N}_{DETECT}}{N_{TRUE}} $$

### Mappability assessment

We used the GEM library [11] to calculate 50mer and 100mer mappability tracks for the GRCm38 mouse genome (−e 0). We then used BEDTools [16] coverage to define mappable regions within the 3′ UTR set published by Herzog et al. [4] to calculate 3′ UTR mappability fractions 3′ UTRs. The same procedure was applied to RefSeq exons (obtained on May 2, 2016) mapped to Entrez genes for exon mappability fractions (note: these also include 3′ UTRs). Entrez genes were mapped to 3′ UTRs and only 3′ UTRs with a mappability fraction below 90% were analyzed.

### Datasets

For in silico validations with simulated datasets, we used the set of mESC 3′ intervals by Herzog et al. [4] as reference. The datasets simulated during this study are listed in Table 2.

For validation, we used real SLAMseq data generated by performing 4SU pulse-chase experiments in mESCs (GEO accession: GSE99970) [4]. The subsets used during this study are listed in Table 3.

For validation of the multimapper recovery strategy, we used RNA-seq data from the same mESC line (GEO accession: GSE99970, samples: GSM2666840-GSM2666842) [4].

### Additional information

Supplementary information, Figures and Tables referenced in this study are provided as .pdf file DUNK_SI.

### Genomic DNA sequencing of AN3–12 cells

AN3–12 mouse embryonic stem cells [12] were lysed in Lysis buffer (10 mM Tris, pH 7.5; 10 mM EDTA, pH 8; 10 mM NaCl; 0.5% N-Laurosylsarcosine) and incubated at 60 °C overnight. DNA was ethanol precipitated using 0.2 M NaCl and the DNA was resuspended in 1x TE. Isolated gDNA was phenol-chloroform purified followed by ethanol precipitation. 2 μg of purified gDNA was sheared in 130 μl of 1xTE in a microTUBE AFA Fiber Crimp-Cap (6 × 16 mm) using the E220 Focused-ultrasonicator (Covaris®) with the following settings: 140 W peak incident power, 10% Duty Factor, 200 cycles per burst, 80 s treatment time. The sheared DNA was bead-purified using Agencourt® AMPure® XP beads (Beckman Coulter) to select fragments between 250 and 500 nt. DNA library preparation was performed using NEBNext® Ultra™ DNA Library Prep Kit for Illumina® (NEB) and the library was sequenced in the paired-end 50 mode on a HiSeq 2500 instrument (Illumina).

## Additional file

Additional file 1: Supplementary information, figures and tables. (PDF 6280 kb)

## Abbreviations

4SU: 4-thiouridine
CPM: Counts-per-million
DUNK: Digital Unmasking of Nucleotide conversions in K-mers
mESC: mouse embryonic stem cell
PCA: Principal component analysis
SLAMseq: Thiol (SH)-linked alkylation for the metabolic sequencing of RNA
SNP: Single Nucleotide Polymorphism
T > C: Thymine-to-cytosine
TPM: Transcripts-per-million
UTR: Untranslated region
