# Supplementary material for: Quantification of experimentally induced nucleotide conversions in high-throughput sequencing datasets
Source: BMC Bioinformatics. 2019 May 20;20:258. doi: 10.1186/s12859-019-2849-7 (PMC6528199; doi:10.1186/s12859-019-2849-7)
Supplement: Supplementary file 1 — Supplementary information, figures and tables. (PDF 6280 kb) [file 12859_2019_2849_MOESM1_ESM.pdf]

Supplementary information

Quantification of experimentally induced nucleotide conversions in high-throughput sequencing datasets

Tobias Neumann[1*], Veronika A. Herzog[2], Matthias Muhar[1], Arndt von Haeseler[3,4], Johannes Zuber[1,5], Stefan L. Ameres[2], and Philipp Rescheneder[3*]

[1]Research Institute of Molecular Pathology (IMP), Campus-Vienna-Biocenter 1, Vienna BioCenter (VBC), 1030 Vienna, Austria
[2]Institute of Molecular Biotechnology of the Austrian Academy of Sciences (IMBA), Dr. Bohr-Gasse 3, VBC, 1030 Vienna, Austria
[3]Center for Integrative Bioinformatics Vienna, Max F. Perutz Laboratories, University of Vienna, Medical University of Vienna, Dr. Bohrgasse 9, VBC, 1030 Vienna, Austria
[4]Bioinformatics and Computational Biology, Faculty of Computer Science, University of Vienna, Waehringerstrasse 17, A-1090 Vienna, Austria
[5]Medical University of Vienna, VBC, 1030 Vienna, Austria

1. BAM/SAM tags

TC:i:

Number of T>C mismatches in a read (A>G if read is on reverse strand)

RA:Z:

Comma-separated integer array, each position marking a specific conversion type.

Read						
		A	C	G	T	N
Reference	A	0	1	2	3	4
	C	5	6	7	8	9
	G	10	11	12	13	14
	T	15	16	17	18	19
	N	20	21	22	23	24

MP:Z:

Comma-separated array of mismatch positions, each position 3 colon-separated values in the format of <type>:<read position>:<reference position> where type is the same as in the RA:Z tag.

2. Count file columns

Column	Content
Chromosome	Chromosome on which the 3’ interval resides
Start	Start position of the 3’ interval (0-based)
End	End position of the 3’ interval (exlusive, 0-based)
Name	Name or ID of the 3’ interval
Length	Length of the 3’ interval
Strand	Strand of the 3’ interval
ConversionRate	ConversionsOnTs / CoverageOnTs for the given 3’ interval
ReadsCPM	Number of reads that mapped to the 3’ interval normalized by library size of retained reads after filtering (counts per million CPM)
Tcontent	Number of Thymines within the 3’ interval

CoverageOnTs	Cumulative coverage on each Thymine of the 3’ interval
ConversionsOnTs	Cumulative number of T>C conversions in the 3’ interval
ReadCount	Number of reads mapping to the 3’ interval
TcReadCount	Number of reads mapped to the 3’ interval with at least *k* T>C conversions (T>C reads)
multimapCount	Number of retained reads considered as multimappers mapping to the 3’ interval
ConversionRateLower	Lower bound confidence interval for 3’ interval (not used)
ConversionRateUpper	Upper bound confidence interval for 3’ interval (not used)

3. Visualization & Reproducibility

SLAM-DUNK comes with a broad panel of visualizations and diagnostic plots to evaluate data quality (QC) on a per-sample basis:

Name	Description	MultiQC
**utrrates**	Summary boxplots of each normalized conversion type over all 3’ intervals	x
**rates**	Barplots of normalized conversion rates over all reads	x
**tcperreadpos**	Mutation rates in percent along read positions, separated into non-T>C mutations and T>C conversions	x

**tcperutrpos**	Mutation rates along the last 200bp of each 3’ interval in percent, separated into non-T>C mutations and T>C conversions	X
**snpeval**	*barcode-plot* of the distribution of T>C SNPs along the ranked upper quartile of T>C containing reads within 3’ intervals	

4. Runtime & Memory usage

We benchmarked SLAM-DUNK with 10 CPU threads on an 4x AMD Opteron 6348 openSUSE 13.2 machine with 512 GB memory. As dataset, we used the 21-sample mESC time course (excluding no 4SU samples) with a mean number of ~20M reads per sample.

The slamdunk all command finished within 8 hours for all 21 samples and used a maximum of 10 GB of main memory. Mapping and conversion to BAM were the most time-consuming steps taking 63% of the overall runtime. Filtering and SNP calling took 27% and 5% respectively. Due to the SAM-tag based implementation counting is with 3% of the overall runtime the least time consuming step of the SLAM-DUNK pipeline.

Supplementary Figures

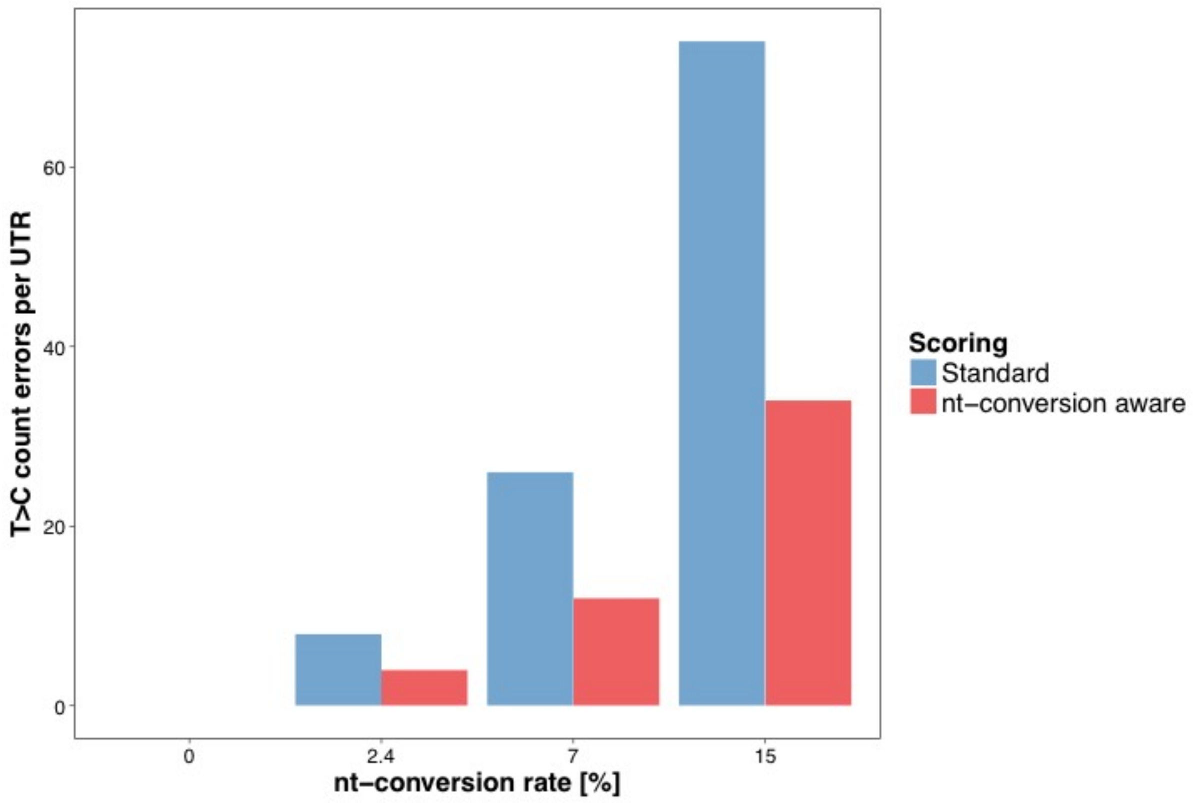

Supplementary Figure S1:
Evaluation of nucleotide conversion aware scoring vs naïve scoring during read mapping: Absolute errors of simulated vs called nucleotide conversion calls are depicted on simulated data with 100 bp read length increasing nucleotide conversion rates at 100x coverage.

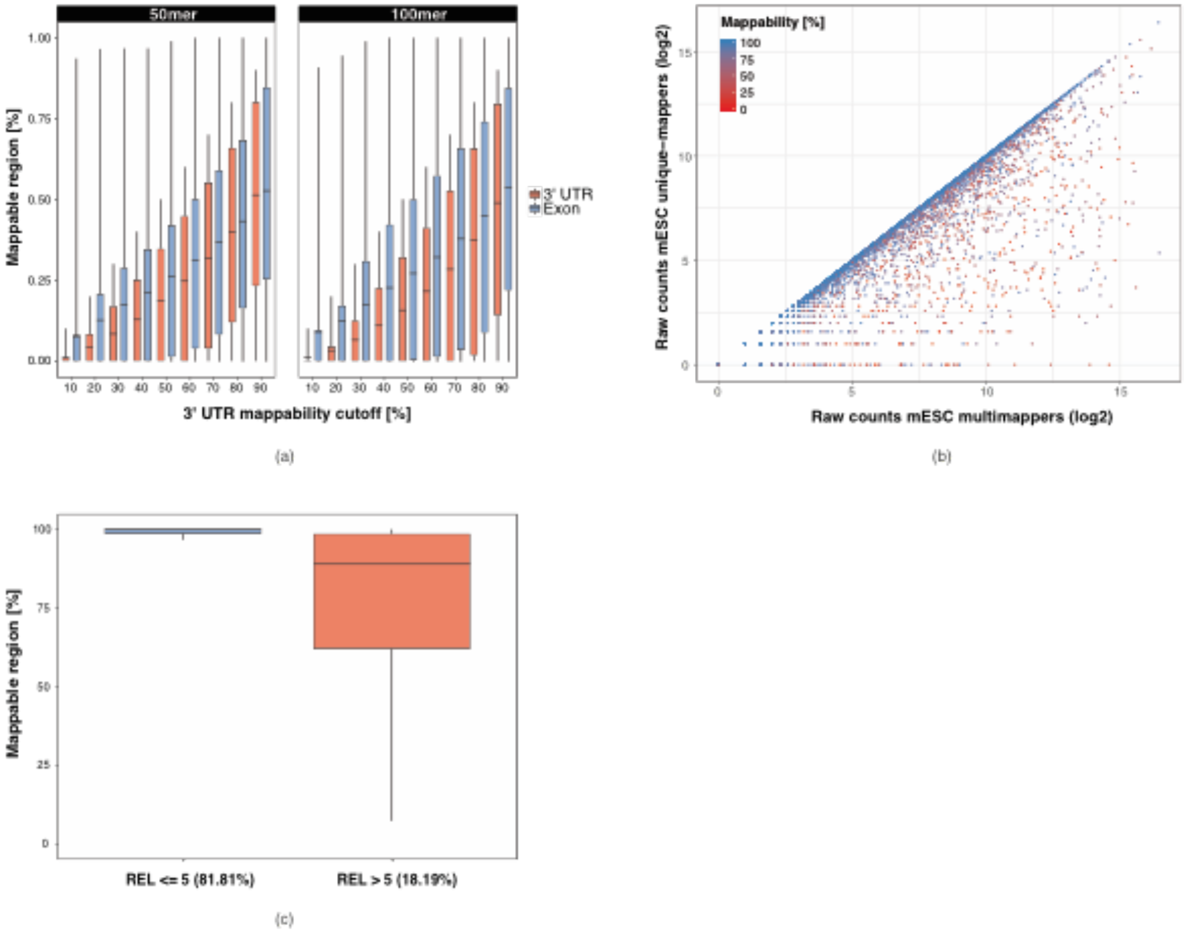

Supplementary Figure S2:

Mappability and correlation with multi-mapper recovery: (a) Mappability percentage distribution within 3’ UTRs and corresponding mappability percentage within all exons (% of total sequence) for 50 and 100mers. Y-axis shows the 25-75 quantile boxplots with indicated mean with increasing 3’ UTR mappability percentage cutoffs for genes up to 90% mappability. (b) Mappability percentages negatively correlate with relative-errors of genes when comparing unique-mappers vs multi-mapper recovery (Spearman’s rho: 0.344, p-value < 2.2e-16). (c) Distribution of mappability percentages in the class of genes with a relative error > 5% is greatly reduced compared to genes with a lower relative error (Mean mappability 77% vs 97%, p-value < 2.2e-16).

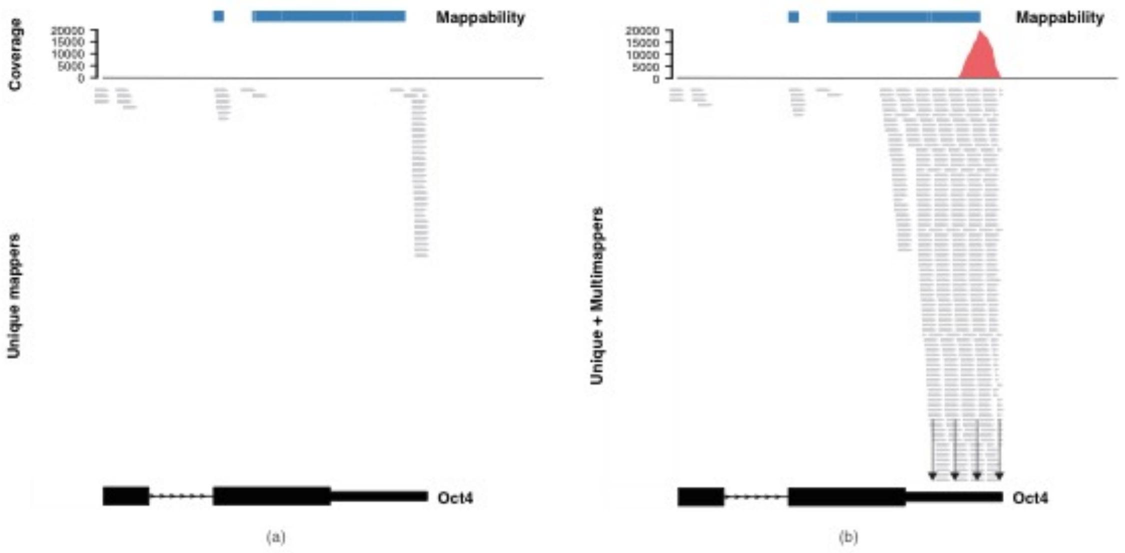

Supplementary Figure S3:

(a) Read coverage for Oct4 when discarding multimapping reads in a representative no 4SU sample. Blue bars indicate unmappable regions (Mappabability < 1) when using 50 bp reads. (b) Read coverage for Oct4 using DUNK’s multimapper recovery.

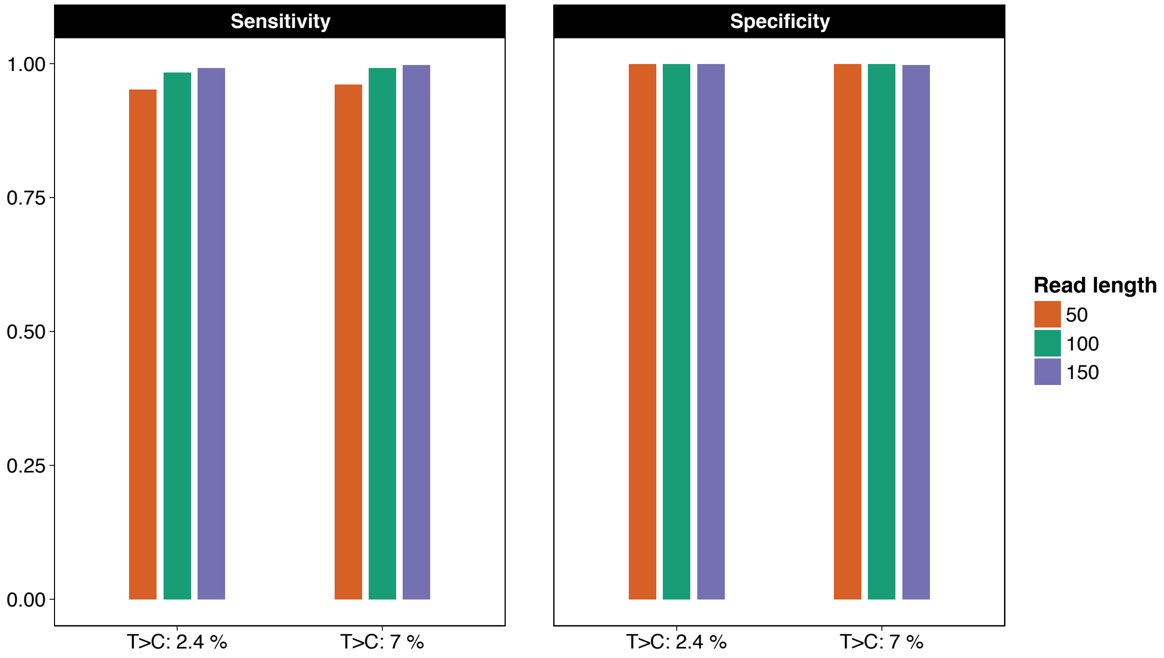

Supplementary Figure S4:

Sensitivity and specificity of SLAM-DUNK on simulated T>C conversion containing reads vs recovered T>C containing reads for read lengths of 50, 100 and 150 bp and nucleotide conversion rates of 2.4 and 7 %.

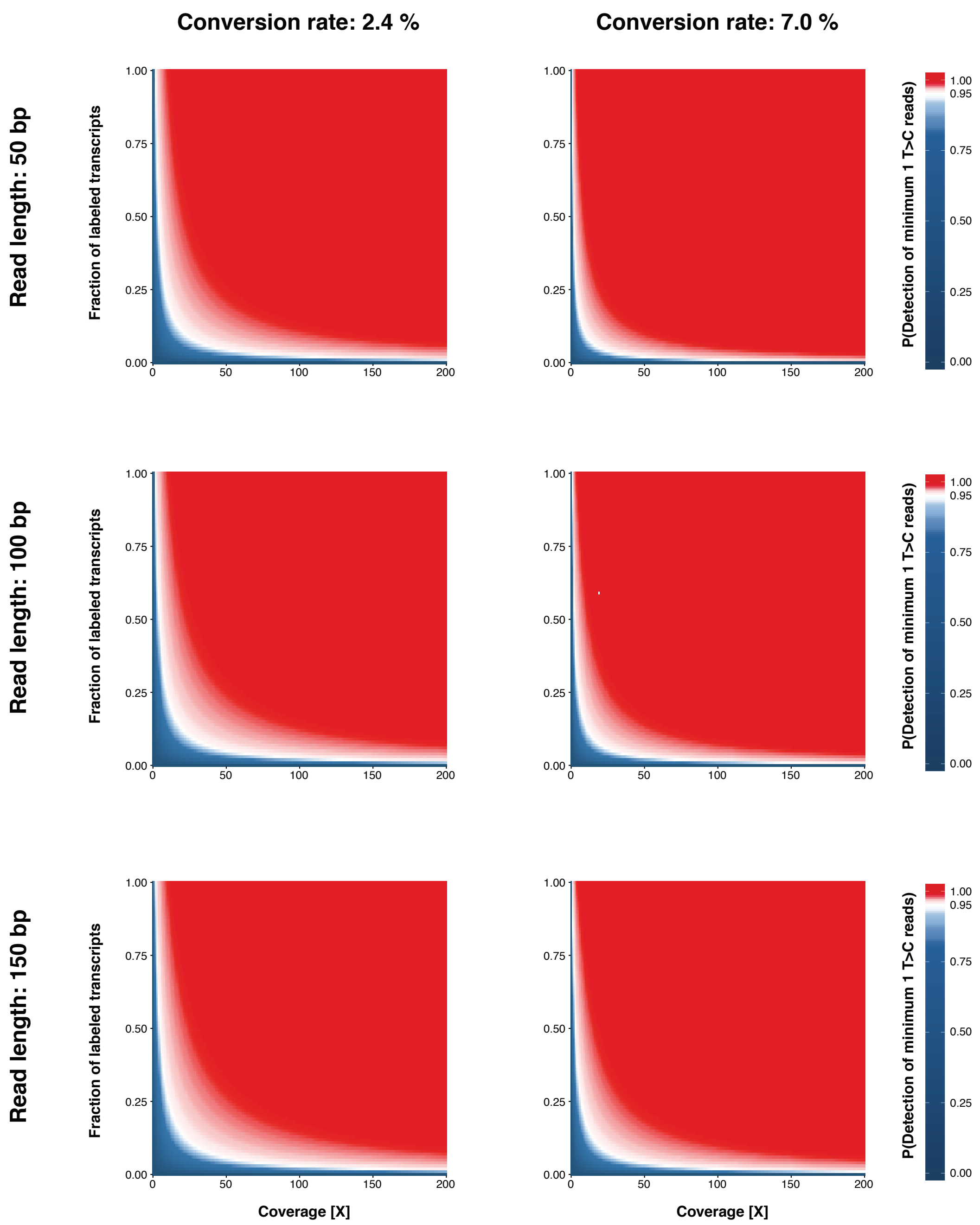

Supplementary Figure S5:

Heatmaps of the probability of detecting at least one read originating from a labeled transcript from a given fraction of labeled transcripts and coverage, for fixed conversion rates of 2.4 and 7% and read lengths of 50, 100 and 150 bp. White color code marks the 0.95 probability boundary.

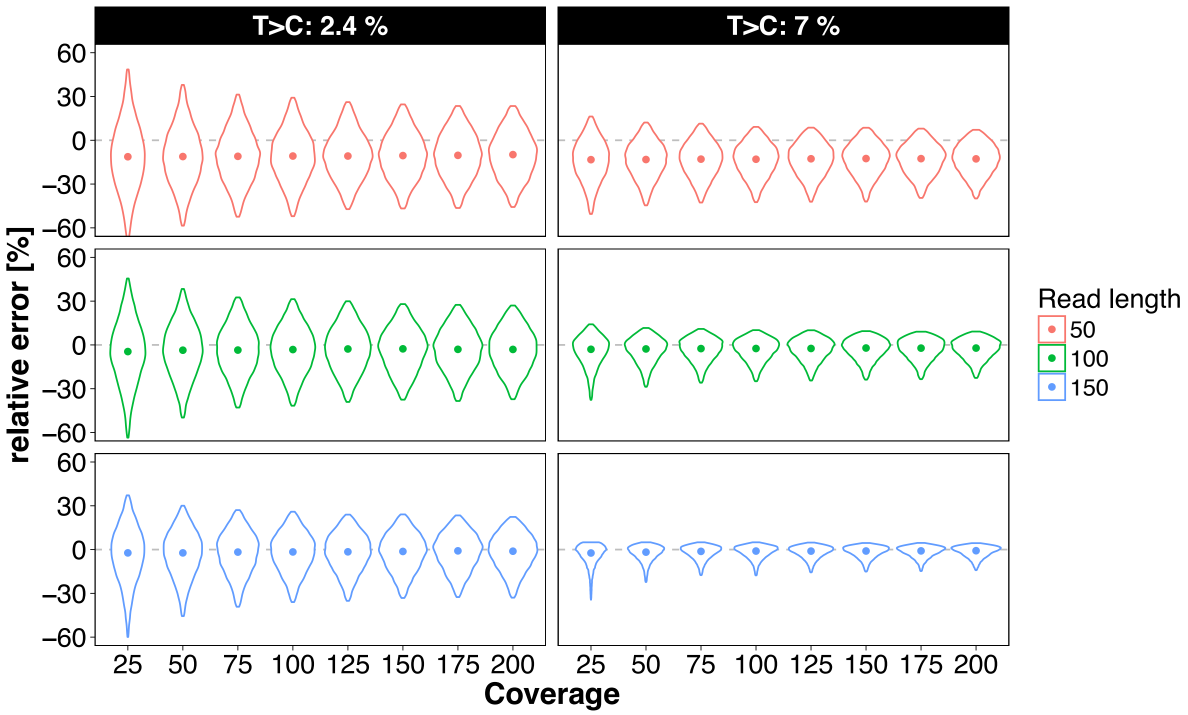

Supplementary Figure S6:

Distribution of relative errors of read-based *fraction of labeled transcripts* estimates for T>C conversion rates of 2.4 and 7 % and sequencing depth from 25 to 200x.

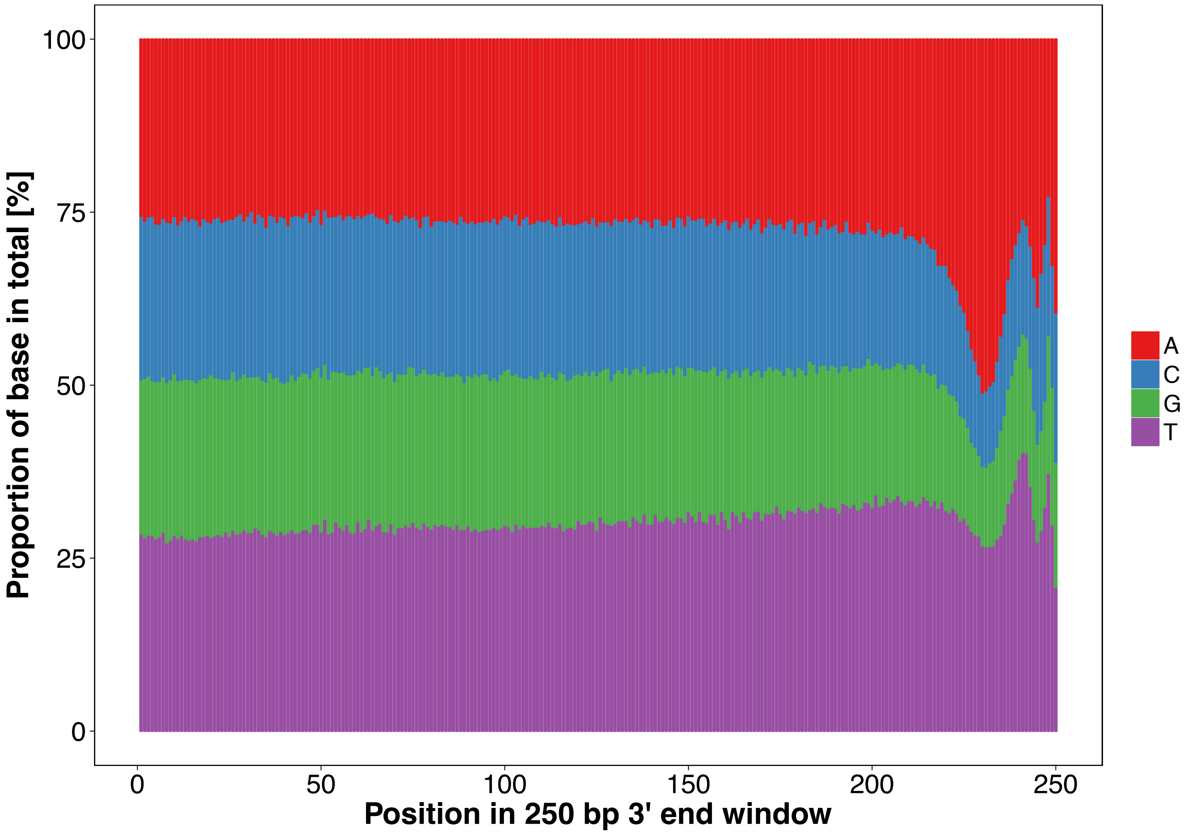

Supplementary figure S7:

Base composition along the last 250bp upstream of the 3’ end of 28,568 published 3’ end counting windows.

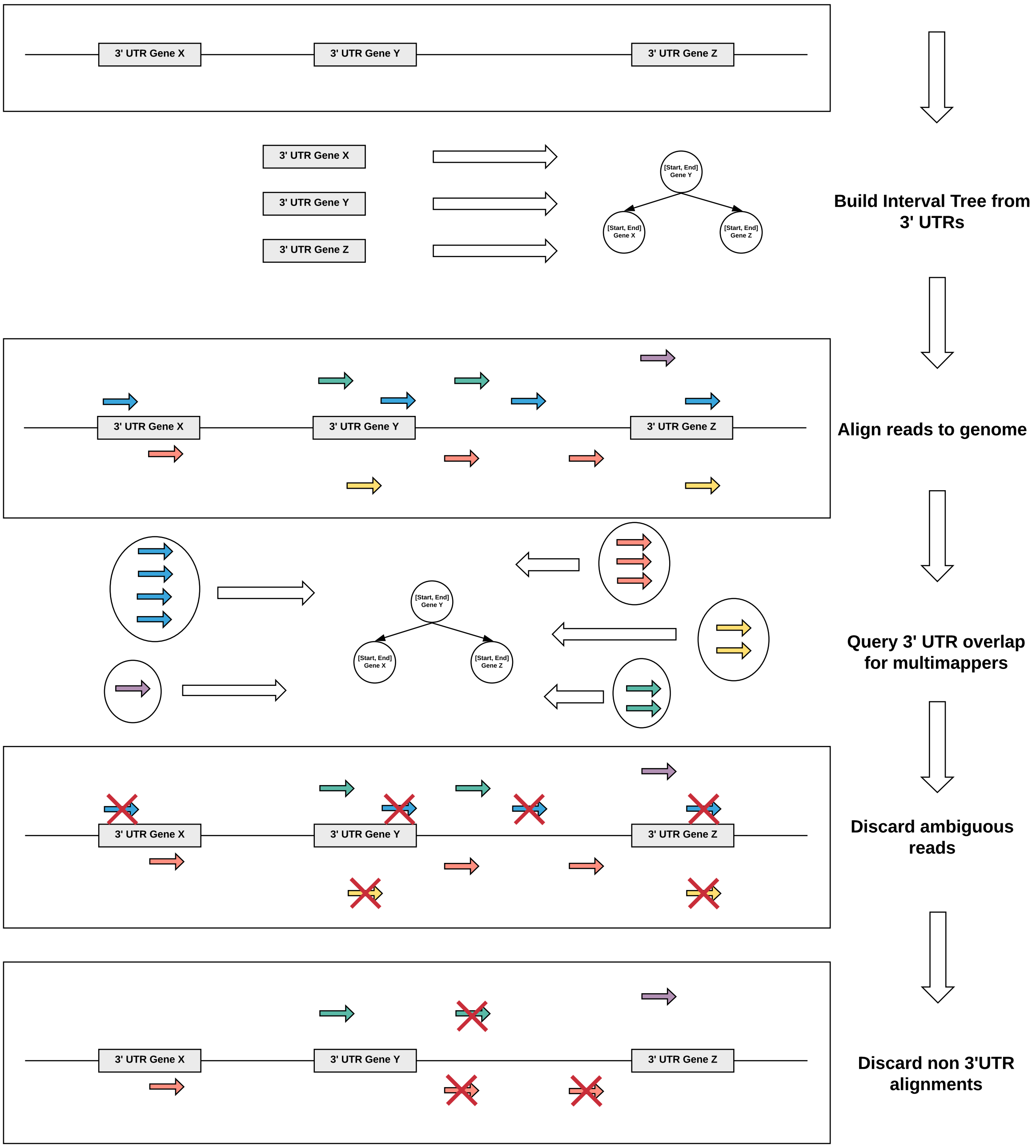

Supplementary Figure S8:

Workflow of multimapper recovery strategy: First, an Interval Tree is created from a given set of intervals (3' UTRs). Then, all alignments of multimapping reads are checked for overlaps with those intervals. Ambiguous reads i.e. multimapping reads with alignments to more than one distinct interval are discarded. Last, alignments of multimappers outside of these intervals are discarded and one distinct alignment within an interval is chosen randomly, if present.

Supplementary Tables

Conversion rate	Read length	Fraction of labeled transcripts	Detection probability > 0.95
**2.4%**	50 bp	0.1	22 X
		0.2	11 X
		0.5	5 X
		1.0	2 X
	100 bp	0.1	26 X
		0.2	13 X
		0.5	5 X
		1.0	2 X
	150 bp	0.1	29 X
		0.2	14 X
		0.5	6 X
		1.0	3 X
**7.0%**	50 bp	0.1	10 X
		0.2	5 X
		0.5	2 X
		1.0	1 X
	100 bp	0.1	14 X
		0.2	7 X
		0.5	3 X
		1.0	1 X
	150 bp	0.1	19 X
		0.2	9 X
		0.5	3 X
		1.0	1 X

Supplementary Table S1:
Overview of required coverage to detect > 0 reads with T>C conversions from labeled transcripts with a probability > 0.95 for selected conversion rates, read lengths and fraction of labeled transcripts within all transcripts.

Gene	3’ intervals	Total length	T-content [%]	Mappable [%]
Drosha	1	296	21.9	100.0
Hdac1	1	260	22.7	31.4
Pou5f1	1	256	23.0	20.3
Actb	1	251	23.5	65.6
Med25	2	500	25.2	100
Mapk8	2	502	25.7	99.9
Tep1	1	250	26.0	100
Papd7	1	299	27.8	100
Adat2	3	905	28.8	100
Dicer1	2	500	29.4	99.9
Rpl34	1	261	29.5	87.7
Rpl12	1	252	29.8	97.3
Nanog	1	252	30.2	42.8
Smad7	1	250	35.6	99.9
Suz12	1	480	36.3	78.8
Hes1	1	374	38.2	100
Ap5m1	2	564	40.3	100
Sox2	1	261	42.1	100

Supplementary Table S2:

18 manually selected example genes covering different numbers of associated 3’ intervals, T-content and mappability.
